# Semi-field evaluation of the bio-efficacy of two different deltamethrin formulations against *Aedes* species in an outdoor residual spraying study

**DOI:** 10.1016/j.heliyon.2020.e03230

**Published:** 2020-01-22

**Authors:** Nurulhusna Ab Hamid, Siti Nurfadhlina Mohd Noor, John Susubi, Nur Rasyidah Isa, Rohaiyu Md Rodzay, Ainaa Mardia Bachtiar Effendi, Afiq Ahnaf Hafisool, Fatin Atirah Azman, Siti Farah Abdullah, Muhammad Khairi Kamarul Zaman, Nazni Wasi Ahmad, Han Lim Lee

**Affiliations:** a Medical Entomology Unit and WHO Collaborating Center for Vectors, Institute for Medical Research, Ministry of Health, Malaysia, Jalan Pahang, 50588, Kuala Lumpur, Malaysia; b Vector Borne Disease Control Program, Kilu'ufi Hospital, Malaita Province, Solomon Islands; c School of Diploma in Applied Parasitology and Entomology, SEAMEO – TROPMED Regional Center Malaysia, Institute for Medical Research, Ministry of Health, Malaysia, Jalan Pahang, 50588, Kuala Lumpur, Malaysia

**Keywords:** Environmental science, Ecology, Indoor and outdoor residual spraying, *Aedes*, Deltamethrin SC-PE, Deltamethrin WG, Dengue, Vector control

## Abstract

In recent decades, dengue incidence has trended upward worldwide causing urgent needs for new or modified vector control methods. We modified the existing indoor residual spraying (IRS) method by applying insecticide on the outer walls of building structures in an outdoor residual spraying (ORS) study. A semi-field study was conducted to investigate the bio-efficacy of two different deltamethrin formulations: K-Othrine® Polyzone, new polymer-enhanced deltamethrin formulated as a suspension concentrate (SC-PE), and K-Othrine® WG 250, traditional deltamethrin formulated as water dispersible granule (WG). The residual bio-efficacy of deltamethrin SC-PE was compared to deltamethrin WG on finished cement surfaces applied to the outer walls at the Institute for Medical Research (IMR), Malaysia. Standard WHO cone wall bioassays were adapted to evaluate the effective duration of action of these deltamethrin formulations against susceptible laboratory-reared and wild, free-flying *Aedes aegypti* and *Ae*. *albopictus*. Analyses of bioassay results showed that deltamethrin SC-PE 30 mg/m^2^ has improved longevity in comparison to deltamethrin WG 30 mg/m^2^. Deltamethrin SC-PE 30 mg/m^2^ was effective until week 17 (producing > 80% mortality), surpassing deltamethrin WG 30 mg/m^2^ which only lasted until week 10. This was supported by post-hoc test analyses which demonstrated that deltamethrin SC-PE 30 mg/m^2^ produced the highest mean of mortality in laboratory-reared *Aedes* species and the wild *Ae. albopictus*. However, the effective duration of action of deltamethrin SC-PE (17 weeks) was less than the recommended period by WHO (6 months) but was reasonable given that the spraying was undertaken outdoor. This preliminary data could be of use for the deployment of locally adapted ORS operation in controlling dengue.

## 1 Introduction

Dengue is a mosquito-borne viral disease of humans affecting the economy and health of communities worldwide. Four distinct serotypes of dengue virus (DENV-1–4) exist which can cause dengue fever (DF) and severe dengue or dengue hemorrhagic fever (DHF). The virus is transmitted among humans through the bite of an infected female of the primary dengue vector, *Aedes aegypti* or the secondary vector, *Ae. albopictus* (WHO, 2009). In recent decades, dengue incidence has increased dramatically from 2.2 million in 2010 to 3.2 million globally in 2015 due to rapid and unplanned urbanization, increased human mobility and trade, viral evolution such as vector resistance to insecticides, and climate change (Murray et al., 2013; WHO, 2017). Since no efficacious, safe and cost-effective vaccine and specific anti-viral treatment against dengue is presently available, current efforts to reduce dengue transmission have focused primarily on vector control through combinations of biological-, gene-, mechanical-, and insecticide-based tools (Lee et al., 2015).

Indoor residual spraying (IRS) is a common insecticide-based tool for vector control intervention. IRS involves the application of a long-lasting residual insecticide to the interior of house walls, eaves, and ceiling where potential vectors might encounter the insecticides while resting on the surfaces (WHO, 2015a). Historically, the IRS was introduced as a vector control method against the malaria vector in 1945 (Giglioli et al., 1974; Gabaldon, 1983). IRS was later used during the Global Malaria Eradication Program (1955–1969) and has succeeded in eliminating malaria from Europe, America, and parts of Asia (Wernsdorfer, 1980; Carter and Mendis, 2002; Tanner and de Savigny, 2008). While IRS has proved to be successful in reducing malaria transmission, evidence on the potential robustness of the IRS to reduce dengue burden is gradually emerging (Paredes-Esquivel et al., 2016; Vazquez-Prokopec et al., 2017a, 2017b). Research in Peruvian Amazon showed that the application of the IRS has significantly reduced the adult index from 18.5 to 3.1 at four weeks post-intervention (Paredes-Esquivel et al., 2016). In Cairns, Australia, targeted IRS was combined with location-based contact tracing has reduced the probability of dengue transmission by 86–96% in comparison to unsprayed control (Vazquez-Prokopec et al., 2017b).

The residual efficacy and persistence of the IRS insecticides play key roles for effective IRS operation. The acceptable WHO threshold for IRS residual activity is to exceed 80% mortality rate (WHO, 2006). There are currently only a few WHO-approved IRS insecticides. They fall into four major classes of IRS insecticides: carbamates, organochlorines, organophosphates, and pyrethroids (WHO, 2015b). Of all the classes, pyrethroids are the most widely used owing to its low mammalian toxicity, relatively inexpensive and long-lasting activities (WHO, 2011). Deltamethrin is an alpha-cyano pyrethroid insecticide of type II group, which kills insects on contact and through digestion, and was first described in 1974 (Elliott et al., 1974). Deltamethrin residual spraying has shown promising results by producing relatively high residual efficacy when tested in several countries in America, Africa, and Asia (Rozilawati et al., 2005; Raeisi et al., 2010; Oxborough et al., 2014; Paredes-Esquivel et al., 2016).

The WHO Pesticide Evaluation Scheme (WHOPES) has recommended the use of deltamethrin water dispersible granule (WG) and deltamethrin polymer-enhanced suspension concentrate (SC-PE) for IRS at a dosage of 20–25 mg/m^2^. According to WHO, the duration of effective action of deltamethrin WG is 3–6 months, whereas, for deltamethrin SC-PE, a duration of 6 months of residual action is proposed (WHO, 2015b). Deltamethrin WG gives a fine suspension in water and is best suited on porous surfaces, while deltamethrin SC-PE is a new alternative formulation which has an extended residual effect through the property of polymer adjuvant that protects the active ingredient deltamethrin against chemical abrasion, alkaline, and rainfall (WHO, 2014). A few studies have tested the two different deltamethrin formulations and found that SC-PE outperformed WG in terms of residual efficacy and longevity (Kijlstra et al., 2014; Oxborough et al., 2014; Dunford et al., 2018).

The WHO has outlined the application of IRS intervention within the Integrated Vector Management (IVM) framework. If the IRS is properly applied through adequate national control programs, the IRS will be optimally deployed and produced a sustainable vector control (WHO, 2015a). At present, we sought to optimize the deployment of the IRS operation that is tailored to the Malaysia context, parallel with the WHO agenda for effective locally adapted vector control measures (WHO, 2017). Owing to the lack of acceptance of homeowners to allow spraying inside houses, we wanted to apply deltamethrin insecticide on the outer walls of houses. We used a modified approach of the IRS termed the ‘outdoor residual spraying (ORS)’ as suggested by Rozilawati et al. (2005), which agrees with the nature of the spraying location. However, we lack data on the residual bio-efficacy and persistence of the deltamethrin WG and the new deltamethrin SC-PE. In this semi-field study, we investigated and compared the residual bio-efficacy of two deltamethrin formulations (WG and SC-PE) at two dosages (25 mg/m^2^ and 30 mg/m^2^) against pyrethroid susceptible strains of laboratory-reared and wild, free-flying *Ae. aegypti* and *Ae. albopictus*. Their residual performance was evaluated on painted cement walls by examining the mosquito mortality in standard WHO cone wall bioassays.

## 2 Methods

### 2.1 Mosquito species

*Aedes* mosquitoes used in this study were laboratory-reared strains and wild, free-flying *Ae. aegypti* and *Ae. albopictus.* The laboratory-reared *Aedes* species were obtained from the Insectarium of the Medical Entomology Unit, Institute for Medical Research (IMR), Kuala Lumpur. The wild *Aedes* species were collected from Johor Bahru, Malaysia via ovitrap surveillance. Ovitraps (300 ml black plastic containers, internal diameter 7.0 cm and height 9.0 cm, equipped with oviposition paddle) containing mosquito larvae were collected and transported back to Johor Bahru District Health Office. The ovitrap content along with the paddle was poured into labeled plastic containers. Beef liver powder (Difco Laboratories, MD, USA) was added to the ovitrap content. Fourth instar larvae were identified and separated into *Ae. aegypti* and *Ae. albopictus* by using established taxonomy keys (Pratt et al., 1964; Parija, 2014) under a compound microscope (Nikon Eclipse® E100, Japan) and were later brought to Insectarium of the Medical Entomology Unit, IMR for breeding. To obtain eggs, female mosquitoes were starved for 12 h prior to blood feeding. Mice were placed inside cylinder cages and were left overnight for the mosquitoes to obtain a full blood meal. After four days, moist filter papers were placed in the cages to allow female mosquitoes to lay eggs. The eggs were later hatched and reared to F4 adults which were used for the cone wall bioassays.

### 2.2 Formulation and application of insecticides

The study was conducted between July 2018 until November 2018 with 20 weeks of data collection. The insecticides used were deltamethrin WG (K-Othrine® WG 250, active ingredient: 250 g/kg deltamethrin, Bayer Crop Science, Monheim am Rhein, Germany) and SC-PE (K-Othrine® Polyzone, active ingredient: 62.5 g/L deltamethrin, Bayer Crop Science, Monheim am Rhein, Germany), which were prepared at two dosages, 25 mg/m^2^ and 30 mg/m^2^ based on the manufacturer's instructions. For the sake of brevity, we abbreviated deltamethrin WG 25 mg/m^2^ to delta WG 25, deltamethrin WG 30 mg/m^2^ to delta WG 30, deltamethrin SC-PE 25 mg/m^2^ to delta SC-PE 25 and deltamethrin WG 30 mg/m^2^ to delta SC-PE 30. The comparisons between deltamethrin WG and SC-PE are summarized in Table 1.

**Table 1:** Comparisons between the two different deltamethrin formulations used in the study.

Formulation	Water dispersible granule (WG)	Polymer-enhanced suspension concentrate (SC-PE)
Description	The insecticide forms a fine suspension after disintegration and dispersion in water (WHO, 2014).	The insecticide forms crystalline particles after dilution in water, which is finer than deltamethrin WG (WHO, 2014; WHO, 2015a).
Advantages	Cheaper than deltamethrin SC-PE, effective on porous surfaces (WHO, 2015a; Dunford et al., 2018).	Has extended residual activity due to the polymer-enhanced property, effective on cement and painted surfaces (Dunford et al., 2018).

The hand compression sprayer (Hudson X-Pert, Chicago, IL, USA) with a flat fan nozzle (Teejet 8002, Spray Systems Co., Bessemer, AL, USA) was calibrated prior to insecticide application as described in WHO (2007) guidelines. The application process was carried out by trained staffs and the appropriate safety procedures were taken as recommended by WHO (2007). Four designated finished cement walls at the IMR, Kuala Lumpur (N 03°10.170′, E 101°42.011’), which represent the four different treatments were sprayed with insecticides at a 6.35 m (wide) x 3 m (height). We decided to apply on painted cement walls because most of the urban houses are constructed from bricks plastered with cement and have painted surfaces. The walls chosen were not directly exposed to sunlight and rainfall.

### 2.3 WHO cone wall bioassays

WHO cone wall bioassays were conducted on sprayed finished cement walls according to WHO guidelines with a few modifications (WHO, 2013). The first bioassay was conducted a week after spraying and repeated at weekly intervals for a period of 5 months. Standard WHO bioassay cones (conical chamber) were firmly and randomly positioned onto the treated wall in a vertical position using masking tape. Bioassays were carried out in the morning (9:00–11:00 am) when the conditions were most suitable (temperature below 30 °C). Ten adult female mosquitoes (sucrose-fed, 3–5 days old, non-blood fed) were carefully introduced into the bioassay cones through the aperture using the battery-operated aspirator. Three technical replicates of 10 mosquitoes were prepared for each *Aedes* species (a total of 120 mosquitoes per treatment). The bioassay cone apertures were plugged with cotton balls and subsequently covered with black cloths. The knockdown time was observed for 30 min at a 1-minute interval. After exposure, the mosquitoes were aspirated out and transferred to clean paper cups. The mosquitoes were held at 27 ± 2 °C with 75 ± 10% relative humidity and were sugar-fed. The mortality was recorded 24 h after testing. The definitions of adult mosquitoes as knocked down or dead were as described in WHOPES (2012). Bioassays were performed 20 times in a period of 5 months and discontinued when the mosquito mortality dropped below the 50% threshold. The bioassays were also conducted on the untreated wall acting as an untreated control.

### 2.4 Data analysis

Data for the weekly bioassays were expressed as percentage knockdown after 30 min and percentage mortality after 24 h, calculated separately for each *Aedes* species and treatment. One-way multivariate analysis of variance (MANOVA) followed by the post-hoc test was carried out to determine if there is a significant difference in mortality between the different treatments for each *Aedes* species. The relationships between time (weeks) and mortality were analyzed using log-week probit regression (Finney, 1971; WHO, 2006). Knockdown and mortality results were used for the probit regression analysis to estimate knockdown time (KD) and lethal time values (LT_50_ and LT_90_), respectively. KD referred to the time to cause knockdown, while LT_50_ and LT_90_ referred to the time taken for the insecticides to cause 50% and 90% of mosquito mortality, respectively. All statistical analyses were conducted using IBM Statistical Package for Social Science software (SPSS) version 20.0 (IBM, 2011).

### 2.5 Ethics statement

The study was approved by the Institute for Medical Research Committee (JPP-IMR) and National Institutes of Health Malaysia (JPP-NIH), and registered with the National Medical Research Register (NMRR-13-921-17915). This study met the ethical standard of the Medical Research and Ethics Committee (MREC) (Ref No: KKM/NIHSEC/800–2/2/2JldP13905).

## 3 Results

The duration of deltamethrin WG and SC-PE residual efficacy against laboratory-reared and wild, free-flying *Ae. aegypti* and *Ae. albopictus* were investigated on cement substrate walls using standard WHO cone wall bioassays in an ORS semi-field study. Mosquitoes were exposed to deltamethrin WG and SC-PE at two dosages: 25 mg/m^2^ and 30 mg/m^2^. 30 min knockdown (KD30) was recorded and mortality was assessed 24 h after exposure.

**Figure 1:**
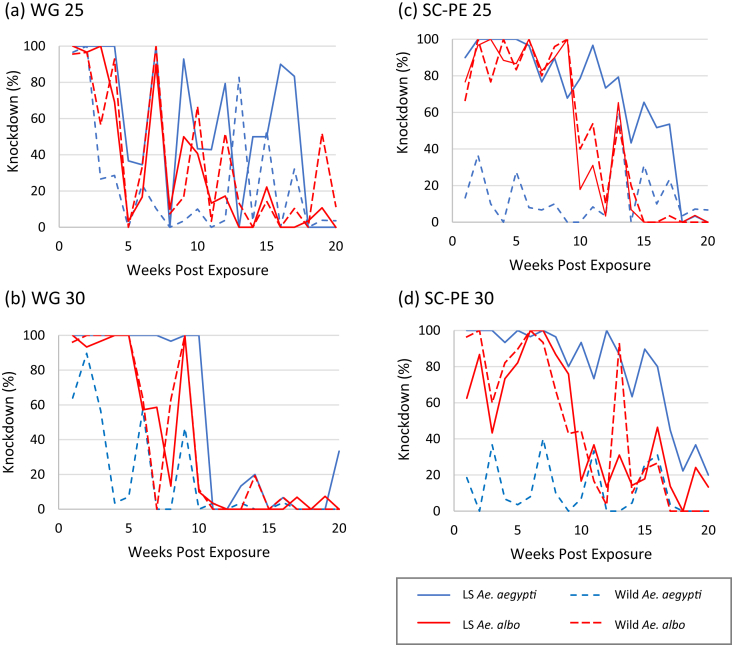
Percentage knockdown 30 min post-exposure (KD30) for LS and wild *Ae. aegypti* and *Ae. albopictus* exposed to treated wall sprayed with (a) delta WG 25, (b) delta WG 30, (c) delta SC-PE 25, and (d) delta SC-PE 30. Abbreviation: LS – laboratory-reared strain.

Bioassay results produced inconsistent knockdown percentage weekly but the overall trend showed that all wall treatments produced KD30 of more than 50% at different stages of post spraying (Figure 1a, b, c, d). This suggested that the residual spraying was knocking down mosquitoes but to different degrees. It was rather unexpected that delta WG 25 treated wall showed knockdown percentages of more than 80% until week 17 compared to delta SC-PE 25 which caused knockdown > 80% until week 11 (Figure 1a, c). Delta SC-PE 30 treatment appeared to produce higher knockdown percentage and exhibited a longer knockdown duration in comparison to delta WG 30 treatment (Figure 1b, d). More than 80% knockdown was produced by delta SC-PE 30 treated wall at 16 weeks post-treatment, whereas the > 80% knockdown of delta WG 30 only lasted until week 10 and continued to decline markedly over the next 10 weeks (0–33.33%). Of the *Aedes* tested, wild *Ae. aegypti* showed the lowest KD30 percentages nearly in all weeks for all treatments.

**Figure 2:**
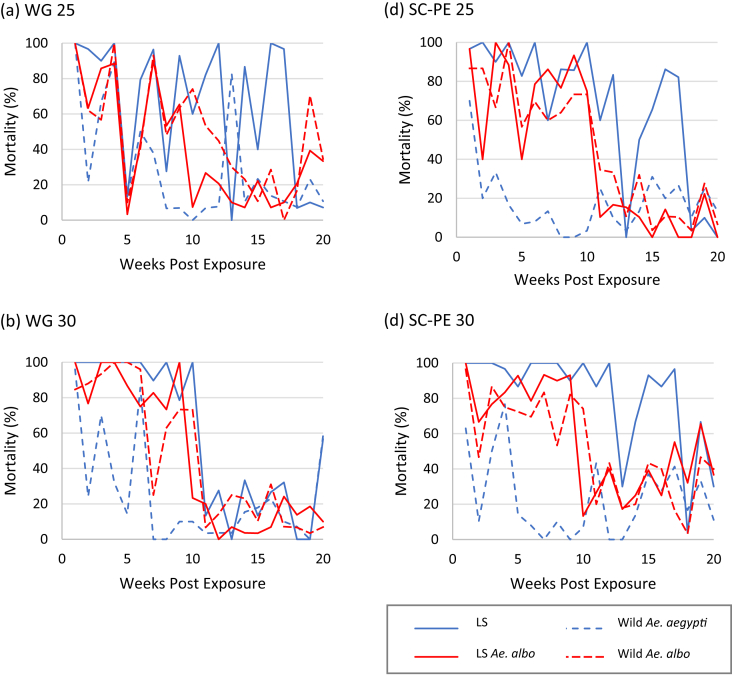
Percentage mortality 24 h post-exposure for LS and wild *Ae. aegypti* and *Ae. albopictus* exposed to treated wall applied with (a) delta WG 25, (b) delta WG 30, (c) delta SC-PE 25, and (d) delta SC-PE 30. Abbreviation: LS – laboratory-reared strain.

Similar to the knockdown rates, huge variations in mortality rates were observed for all treated walls across the tested *Aedes* species during the period of study (Figure 2a, b, c, d). There was 100% mortality for all tested *Aedes* on delta WG 25 treated wall in the initial first week after spraying. Mortality rates remained relatively high until week 4 but suddenly decreased to a very low level (3.33–13.33% across tested *Aedes*) in week 5, and continued to vary until week 20 (Figure 2a). It was worth noting that delta WG 30 has longer residual activity than delta WG 25 as evident from the high mortality rates (above the WHO 80% mortality threshold) for all *Aedes* populations within the first 10 weeks of post spraying (Figure 2b). The walls treated with delta SC-PE 25 and SC-PE 30 induced high mortality rates (> 80%) for laboratory-reared strain *Ae. aegypti* in 12 out of 20 weeks and produced mortality > 80% until week 17 (Figure 2c, d). In comparison, a relatively low level of mortality was generated for wild *Ae. aegypti* on SC-PE 25 and SC-PE 30 treated walls. Mortality in the absence of insecticide (untreated control) was less than 5% throughout the study.

Post-hoc analyses showed that delta SC-PE 30 produced the highest mean of mortality in three *Aedes* species: laboratory-reared strains *Ae. aegypti* (80.3 ± 3.9) and *Ae. albopictus* (55.2 ± 4.0) as well as the wild *Ae. albopictus* (49.3 ± 3.7; Table 2). Delta WG 30 treatment produced the highest mean of mortality in wild *Ae. aegypti* (22.5 ± 3.7). However, it was worth noting that delta SC-PE 30 induced only a slightly lower mean of mortality (22.2 ± 3.1) compared to delta WG 30 (Table 2). Only laboratory-reared strain *Ae. aegypti* has a significant difference in mean of mortality (*p*-value < 0.05) between the four different deltamethrin treatments. No significant differences in the mean of mortality (*p*-value > 0.05) were found for the other tested *Aedes*.

**Table 2:** Comparisons of the mean of mortality for each *Aedes* species and strain between the four different treatments analyzed using the post-hoc test in SPSS Statistics.

*Aedes* species and strain	Treatment	Mean ± SE	95% CI	*p*-value
LS *Ae. aegypti*	Delta WG 25	65.3 ± 4.8	(55.8, 74.9)	0.007*
	Delta WG 30	57.5 ± 5.4	(46.8, 68.2)	
	Delta SC-PE 25	65.3 ± 4.8	(55.8, 74.9)	
	Delta SC-PE 30	80.3 ± 3.9	(72.5, 88.1)	
Wild *Ae. aegypti*	Delta WG 25	17.0 ± 2.5	(12.1, 21.9)	0.359
	Delta WG 30	22.5 ± 3.7	(15.9, 38.0)	
	Delta SC-PE 25	17.0 ± 2.5	(12.1, 21.9)	
	Delta SC-PE 30	22.2 ± 3.1	(16.0, 28.3)	
LS *Ae. albopictus*	Delta WG 25	39.0 ± 5.0	(29.1, 48.9)	0.055
	Delta WG 30	44.5 ± 5.1	(34.4, 54.6)	
	Delta SC-PE 25	39.0 ± 5.0	(29.1, 48.9)	
	Delta SC-PE 30	55.2 ± 4.0	(47.3, 63.1)	
Wild *Ae. albopictus*	Delta WG 25	43.5 ± 4.4	(34.7, 52.3)	0.550
	Delta WG 30	41.0 ± 4.3	(32.3, 49.7)	
	Delta SC-PE 25	43.5 ± 4.4	(34.7, 52.3)	
	Delta SC-PE 30	49.3 ± 3.7	(42.0, 56.7)	

Notes: * indicates there is a significant difference between treatments.

The time required to kill 50% of the *Aedes* population (LT_50_) and 90% of the population (LT_90_) of exposed mosquito was calculated using log-week probit regression. *Aedes* population exposed to delta SC-PE 30 exhibited a range of LT_50_ values of 4.7 days–20.8 weeks and LT_90_ values of 0.06 days–9.0 weeks while delta WG 30 displayed a range of LT_50_ values of 2.4–11.7 weeks and LT_90_ values of 2.1 days–7.1 weeks (Table 3). These results suggested that delta SC-PE 30 is a better formulation than delta WG 30 because it killed 90% of the population in less than a day (as opposed to delta WG 30 which took 2.1 days). Delta SC-PE 30 also lasted longer than delta WG 30 since it was still effective in eliminating 90% of the mosquito population at 8.9 weeks compared with 7.1 weeks for delta WG 30. The same trend was observed when comparing LT_90_ between delta SC-PE 25 and delta WG 25. Delta SC-PE 25 exhibited LT_90_ in less than a day and persisted until 5.0 weeks. In comparison, delta WG 25 showed LT_90_ of 3.9 days and has a shorter residual action (LT_90_ = 2.6 weeks).

**Table 3:** Probit mortality per log time (weeks) regression analyses for each treatment from which LT_50_ and LT_90_ were estimated. The analysis was conducted using SPSS Statistics.

Treatment	*Aedes* species and strain	Lethal time, LT (weeks)		Regression coefficient ± SE
		LT_50_ (95% CI)	LT_90_ (95% CI)	
Delta WG 25	LS *Ae. aegypti*	15.125 (10.426–35.789)	2.632 (0.298–4.743)	1.991 ± 0.219
	Wild *Ae. aegypti*	3.532 (1.715–5.132)	0.553 (0.073–1.276)	0.872 ± 0.164
	LS *Ae. albopictus*	5.981 (4.179–7.721)	1.283 (0.425–2.213)	1.489 ± 0.182
	Wild *Ae. albopictus*	7.617 (4.655–11.259)	0.825 (0.060–1.954)	1.171 ± 0.176
Delta WG 30	LS *Ae. aegypti*	11.730 (10.439–13.009)	7.051 (5.330–8.292)	6.200 ± 0.514
	Wild *Ae. aegypti*	2.375 (0.630–3.994)	0.297 (0.010–0.929)	0.533 ± 0.163
	LS *Ae. albopictus*	8.351 (6.755–9.974)	3.692 (2.215–4.901)	3.333 ± 0.270
	Wild *Ae. albopictus*	7.975 (5.337–10.788)	2.933 (0.925–4.611)	2.661 ± 0.235
Delta SC-PE 25	LS *Ae. aegypti*	13.526 (10.278–20.903)	4.960 (1.750–7.137)	3.328 ± 0.302
	Wild *Ae. aegypti*	0.431 (0.003–1.411)	0.009 (0.000–0.122)	-0.275 ± 0.155
	LS *Ae. albopictus*	6.493 (4.823–8.205)	1.995 (0.895–2.908)	2.032 ± 0.195
	Wild *Ae. albopictus*	7.516 (5.867–9.313)	2.060 (1.011–3.055)	1.997 ± 0.195
Delta SC-PE 30	LS *Ae. aegypti*	20.819 (16.511–38.278)	8.955 (5.166–11.162)	4.612 ± 0.488
	Wild *Ae. aegypti*	0.669 (0.000–2.241)	0.009 (0.000–0.192)	-0.119 ± 0.155
	LS *Ae. albopictus*	11.583 (8.917–16.191)	1.978 (0.667–3.258)	1.776 ± 0.197
	Wild *Ae. albopictus*	8.997 (6.715–12.052)	1.282 (0.367–2.304)	1.445 ± 0.177

## 4 Discussion

WHOPES has recommended an insecticide dosage of 20–25 mg/m^2^ with deltamethrin as the active ingredient for the IRS against malarial vectors (WHO, 2015b). Here, we tested the use of a higher deltamethrin dosage of 30 mg/m^2^ (as well as the recommended dosage of 25 mg/m^2^) as dengue prevention and vector control measure for application in ORS. This decision was made after careful consideration of the spraying locations, which were applied at the exterior walls. Although the walls were shielded by roofs, they may occasionally be subjected to rain splash and sunlight that will reduce insecticide effectiveness. Since insecticide effectiveness wanes over time, a higher dosage of insecticide will hopefully compromise its residual efficacy and durability. ORS could serve as a complementary method with the existing method of space spraying which is routinely applied during dengue outbreaks in Malaysia. The efficacy of the conventional method of space spraying methods is limited for several reasons, i.e. the method is only effective to the adult mosquitoes, its residue can last only a few hours and frequently, the insecticides do not reach or penetrate inside houses (Vythilingam and Wan-Yusoff, 2017).

The outcomes of this semi-field study showed that the ORS approach is working but to a lesser extent than the WHOPES recommendation of the duration of effective action for deltamethrin WG (3–6 months) and SC-PE (6 months) (WHO, 2015b). Nevertheless, the effective duration of insecticides tested appeared to be reasonable given that the residual spraying was conducted outdoors. We found that the delta SC-PE 30 was effective until week 17 (> 80% mortality), surpassing delta WG 30 which only lasted until week 10. The effectiveness of delta SC-PE 30 declined substantially for 3 consecutive weeks at week 17, and thus the study was brought to an end. It was conceivable that the higher percentage mortality and increased longevity of delta SC-PE compared to the delta WG were due to the improved formulation of delta SC-PE which has the property of protecting deltamethrin from eroding and the slow release of deltamethrin over a longer period. ORS intervention using delta WG 25 has previously been tested against *Aedes* species and showed fairly good efficacy, but the study only lasted for six weeks (Rozilawati et al., 2005). Another study was later conducted using delta SC-PE 30 in the low-rise and high-rise residences. Bioassay results showed that the mortality percentage for *Aedes* was more than 80% for 16 weeks (Ab Hamid et al., 2019). Research in Tanzania using delta SC-PE showed to effectively cause 100% mortality in *Blattella germanica* for a period of 3 months even after exposure to sunlight, rainfall and daily temperature fluctuations (Kijlstra et al., 2014).

Additionally, the high mean mortality produced by delta SC-PE in this study probably resulted from the suitable application of delta SC-PE on cement painted walls. The SC is more effective on finished cement and wood, as well as the oil-based painted surface, while WG performs better on a very porous surface such as mud bricks and concrete walls (WHO, 2015a). A recent study of deltamethrin used in the IRS also showed that the delta SC-PE produced greater mortality rates on cement walls compared to delta WG (Dunford et al., 2018). The effectiveness of delta WG (above the WHO 80% mortality threshold) on cement walls seemed to be inconsistent ranging from 1 – 5 months when tested in East and West Africa (Dengela et al., 2018). The higher mean mortality generated by delta SC-PE 30 compared to delta SC-PE 25 was most probably because of a higher concentration of deltamethrin in SC-PE 30. Similarly, dosage-related findings from an IRS study in Africa revealed that delta SC-PE 50 has a higher mortality percentage compared to delta SC-PE 25 when tested on a concrete surface (Oxborough et al., 2014).

The wild, free-flying *Ae. aegypti* induced the lowest mean mortality in all treatments which suggested that there was the possibility of insecticide resistance occurring in this major dengue vector. *Ae. aegypti* is typically found in urban areas, and consequently has a higher likelihood of exposure and contact to insecticides such as household insecticides, larvicide and ULV fogging (Teng and Singh, 2001; Chen et al., 2005). Heavy use of these insecticides could increase the selection pressure for resistance to insecticides. Pyrethroid insecticide resistance is widespread in *Ae. aegypti* but is comparatively at a low level for *Ae. albopictus* (Ranson et al., 2010; Vontas et al., 2012). The existence of deltamethrin resistance in wild *Ae. aegypti* has been reported in Johor Bahru, where the mosquitoes were collected (Ishak et al., 2015).

The lack of consistency of knockdown and mortality rates of *Aedes* mosquitoes was probably influenced by several factors that cannot be fully justified in this study. A plausible explanation may involve the degradation of insecticides. The bioassay cones were randomly positioned on the sprayed walls where some parts of the surfaces may be exposed to sunlight and rain splash. Exposure to sunlight can lead to photodegradation while humidity or moisture can cause hydrolysis of the insecticides. The walls may also be subjected to elevated temperatures (thermal degradation) during hot days and microbial attack (biodegradation) (Sibanda et al., 2011). A few studies have shown that the residual activity of pyrethroid insecticides declines upon exposure to sunlight and rain (Peck et al., 2014; Lee et al., 2015). However, pyrethroids were far more stable than other classes of insecticides (organophosphates, carbamates, and DDT) when tested as IRS in the laboratory settings (Sibanda et al., 2011).

The IRS insecticides are contact poisons at which the lethal dose is absorbed by mosquitoes during the resting phase (WHO, 2015a). *Ae. aegypti* is thought to be largely endophilic that rests considerable time indoors (Chadee, 2013; Dzul-Manzanilla et al., 2017). But subspecies *Ae. aegypti formosus* which is found typically in Sub-Sharan Africa has been reported to be mainly exophilic (resting outdoors) (Dickson et al., 2014). Not many studies have been done on the resting behavior of *Aedes* species in Malaysia. A study reported that *Ae. aegypti* mainly rest indoors on temporary objects such as clothing and mosquito nets (Macdonald et al., 1965), while *Ae. albopictus* is categorized as a more exophilic species (Estrada-Franco and Craig, 1995). For effective ORS treatment, mosquitoes should be resting on the sprayed surface at ample time to pick up the lethal dose before or after feeding. We believe that there may be behavior plasticity of *Aedes* occurring in Malaysia where people nowadays tend to spend more time outdoors, shifting their resting behavior to exophily, though further studies are warranted to confirm this. Behavior plasticity has been documented for *Anopheles arabiensis* where the resting behavior changes according to season and temperature (Paaijmans and Thomas, 2011).

## 5 Conclusions

Our results indicated that the performance of the newly formulated delta SC-PE exceeded that of traditionally formulated delta WG on treated cement surfaces. The duration of action of SC-PE, however, was less than the WHO recommendation of the residual period for delta SC-PE. These results could be used by the Ministry of Health, civil societies and private sector to design a strategic plan for a cost-effective and efficient ORS operation, as outlined in the IVM framework. Many factors should be considered before ORS can be implemented such as public engagement, the frequency of spray rounds, household's accessibility and distribution as well as the average surface area to be sprayed. Along with the epidemiological and entomological surveillance activities, a locally adapted and sustainable national dengue program for vector control could potentially reduce the incidence rates of DF and DHF.

## Declarations

### Author contribution statement

Nurulhusna Ab Hamid: Conceived and designed the experiments; Analyzed and interpreted the data; Contributed reagents, materials, analysis tools or data; Wrote the paper.

Siti Nurfadhlina Mohd Noor: Performed the experiments; Analyzed and interpreted the data; Wrote the paper.

John Susubi, Rohaiyu Md Rodzay, Ainaa Mardia Bachtiar Effendi, Afiq Ahnaf Hafisool, Fatin Atirah Azman, Siti Farah Abdullah, Muhammad Khairi Kamarul Zaman: Performed the experiments.

Nur Rasyidah Isa: Performed the experiments; Analyzed and interpreted the data.

Nazni Wasi Ahmad, Lee Han Lim: Conceived and designed the experiments.

### Funding statement

This work was supported by the Ministry of Health, Malaysia (Grant number: NMRR-13-921-17915).

### Competing interest statement

The authors declare no conflict of interest.

### Additional information

No additional information is available for this paper.
